# Incidence of Undifferentiated Pleomorphic Sarcoma (UPS) in the United States

**DOI:** 10.1155/2024/6735002

**Published:** 2024-10-29

**Authors:** Jiemin Ma, Roman Groisberg, Changxia Shao, Wenjun Zhong

**Affiliations:** Merck & Co., Inc., Rahway, New Jersey, USA

## Abstract

The classification of undifferentiated pleomorphic sarcoma (UPS) has been evolving with advances in immunohistochemistry and genomic profiling over the past 20 years. There is a lack of current information on UPS incidence. Due to the lack of designated histology codes for UPS in the Surveillance, Epidemiology, and End Results (SEERs) program, we estimated UPS incidence by three different definitions based on clinical opinions using the 2000–2020 data from 22 registries of the SEER program. The incidence varied widely across the three definitions with 0.06 per 100,000 persons for the least inclusive definition and 0.67 per 100,000 persons for the most inclusive definition in 2016–2020, making it challenging to estimate the exact incidence of UPS. Regardless, all the incidences decreased between 2000 and 2020. Guidelines in UPS diagnosis and classification need to be better implemented in the US.

## 1. Introduction

Undifferentiated pleomorphic sarcoma (UPS), previously known as malignant fibrous histiocytoma (MFH), was a common subtype of soft tissue sarcomas (STS) until the early 2000s [1]. However, over the years, the concept of fibrohistiocytic differentiation has been challenged. It has been found that the phenotype of MFH is more closely aligned with a fibroblast than a histiocyte [2]. In 2013, MFH was removed from the World Health Organization (WHO) classification of STS. Currently, UPS is diagnosed after exclusion of specific lines of differentiation and represents the prototypical storiform and pleomorphic variant of MFH [3].

UPS has been suggested to be among the most common types of STS, but the real incidence of UPS in US is not clear. There is emerging evidence that UPS is uniquely sensitive to immunotherapy. A better understanding of the incidence of UPS is needed to design future clinical trials and assess the impact that this could potentially have on patients. The older name of UPS, MFH, was thought to account for approximately 50% of STS until early 2000, but this entity has been deleted in the WHO classification since 2013 [3]. Hence, there is significant limitation of the historical literature on the incidence of UPS. Therefore, we conducted this analysis to understand the incidence of UPS in the US. The Surveillance, Epidemiology, and End Results (SEERs) Program of the National Cancer Institute (NCI) is an authoritative source of information on cancer incidence in the United States. SEER currently collects and publishes cancer incidence and survival data from population-based cancer registries covering approximately 48.0 percent of the U.S. population. In the most recent histology code list in the SEER program, there are no designated codes for UPS yet. Using MFH as the proxy of UPS, it showed that the proportion of MFH in STS decreased drastically, from 17% in 2002–2006 to 2.7% in 2014–2018 in SEER [4, 5]. This percentage (2.7%) may not reflect the true burden of UPS in the US due to the changing guidelines for STS classification.

Based on input from our clinical experts, UPS could potentially be coded into four other subtypes of sarcomas in clinical practice, including fibrosarcoma NOS (not otherwise specified), fibromyxosarcoma, fascial fibrosarcoma, and myxosarcoma. Fibrosarcomas (including myxofibrosarcomas) are tumors with cells that are reminiscent of normal fibroblasts and usually arise in deep tissues. Some UPS might also be coded as sarcoma NOS. Therefore, we aim to explore the incidence of UPS by including these STS subtypes and estimate the UPS disease burden in all STS using SEER data. The current project aims to address and narrow down the existing gap in incidence of UPS in the US. Our study could not provide the precise estimate on UPS incidence in US due to the lack of designated codes of UPS; however, by implementing multiple ways of estimates, this study can provide our best estimate on the likely range of UPS incidence and explore the impact based on different definitions. The project endeavors will provide tangible solutions that will significantly reduce the gap and pave the way for enhanced UPS epidemiology.

## 2. Materials and Methods

The 2000–2020 SEER22 data were used to estimate the incidence of STS and UPS. STS patients were identified using the histology codes listed in Table 1, and the primary cancer site was restricted to connective, subcutaneous, and other soft tissues (ICD-O-3 site code: C49.0–C49.9). UPS patients were identified using three different definitions (see Table 2).

Crude and age-adjusted (2000 US standard population) incidences of UPS for 2016–2020 were estimated, and annual incidence and proportion of UPS in STS were also estimated from 2000 to 2020 to understand the trends over the years. All analyses were performed using SEER ∗ stat 8.42.

## 3. Results

During 2016–2020, the crude incidence of UPS was 0.06 per 100,000 persons when using MFH only to define UPS, representing 1.8% of all STS (Table 2). The crude incidence and proportion were 0.38 per 100,000, and 11.2% when including fibrosarcoma NOS, fibromyxosarcoma, fascial fibrosarcoma, and myxosarcoma in the second definition and were 0.67 per 100,000 and 20.0% when further including sarcoma NOS.

**Figure 1:**
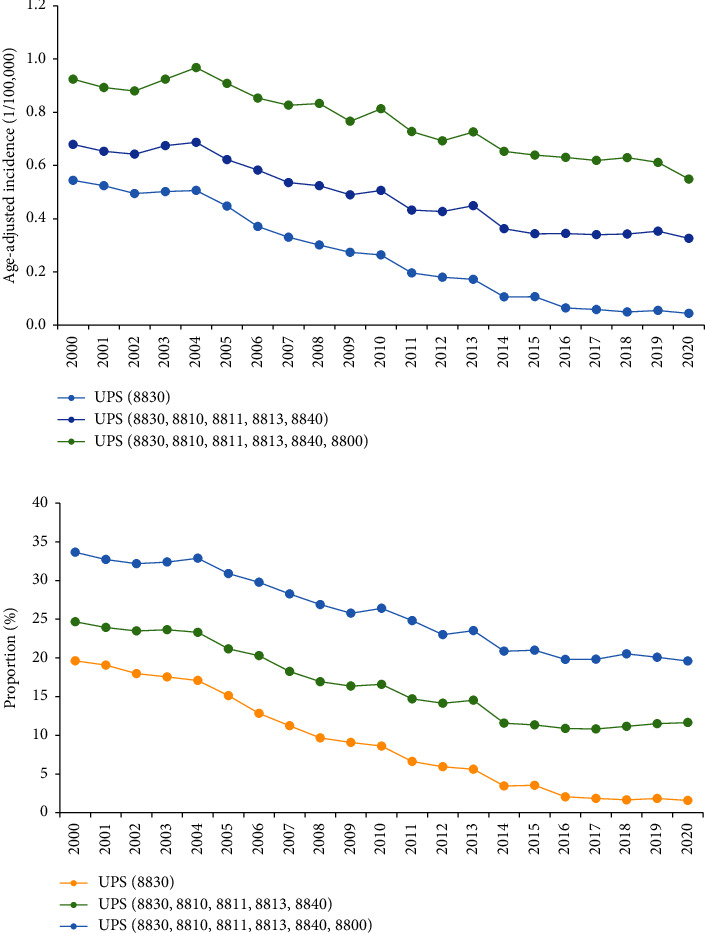
Trend from 2000 to 2020 for (a) age-adjusted incidence of undifferentiated pleomorphic sarcoma (UPS) and (b) UPS proportion among soft tissue sarcomas by different definitions.

During 2000–2020, for all the three UPS definitions, the incidence of UPS was relatively stable in 2000–2004, followed by a steep decrease (Figure 1(a)). The proportion of UPS in STS also had a similar pattern (Figure 1(b)). From 2004 to 2020, in the least inclusive definition, UPS incidence decreased from 0.51 to 0.04 per 100,000, and the proportion of UPS in STS decreased from to 17.1% to 1.6%; in the second definition, UPS incidence decreased from 0.69 to 0.33 per 100,000, and the proportion of UPS in STS decreased from to 23.3% to 11.6%; in the most inclusive definition, UPS the incidence decreased from 0.97 to 0.55 per 100,000 persons and the proportion decreased from 32.9% to 19.6%.

## 4. Discussion

There are little data on the incidence of UPS, and historical literature on MFH may have significant limitation due to evolving STS classification guideline. To our knowledge, this is the first study aiming to understand the burden of UPS in the US population using three different definitions. The three crude incidences would translate into annual new UPS cases of 202 (MFH only), or 1277 (adding fibrosarcoma NOS, fibromyxosarcoma, fascial fibrosarcoma, and myxosarcoma), or 2051 (further adding sarcoma NOS).

Consistent with previous studies, we found that UPS defined as MFH only accounted for 17.1% of total STS in 2004 and the proportion dropped to 1.8% during 2016–2020 [1, 4, 5]. A drop of the proportion of UPS in STS over time is expected because of the advancement of immunohistochemistry and the availability of genetic/molecular markers. Though MFH was removed from the WHO classification since 2013 [6], in SEER, the MFH code is still used and there is no designated histology codes for UPS. Some recent publications continue to solely use MFH to describe UPS [7, 8]. However, it is not clear if it is adequate to use MFH only to reflect the true burden of UPS given the drastic decline in the incidence and proportion in STS. Clinical experts suggested that due to the confusion in UPS definition and classification, some UPS cases could potentially be classified into fibrosarcoma NOS, fibromyxosarcoma, fascial fibrosarcoma, myxosarcoma, and sarcoma NOS. Thus, we estimated the incidences by including these STS subtypes to define UPS. UPS incidence based on the most inclusive definition could serve as the upper limit of the true incidence. The current incidence of UPS in the US is likely higher than 0.06 per 100,000 but probably not higher than 0.67 per 100,000 persons.

A few studies have reported the proportion of UPS in STS. Among 10,000 STS patients from Memorial Sloan Kettering cancer center between 1982 and 2013, UPS accounted for 14% [9]. Another study from France included over 12,000 STS cases from multiple centers between 1980 and 2013 and UPS accounted for 11% [10]. With the improvement in technology to aid in STS classification, the proportion of UPS in STS in current years might be even lower than these reported numbers but probably not as low as 1.8% (our first definition of UPS as MFH only). Our estimate of UPS incidence based on the definition of MFH plus fibrosarcoma NOS, fibromyxosarcoma, fascial fibrosarcoma, and myxosarcoma appears to be closer to the true estimate based on these reported proportions confirmed by pathologists [9, 10]. No matter which definition was used, the incidence of UPS continued to decrease from 2004.

One limitation of our study is the uncertainty of the UPS diagnosis codes used in clinical practice. For example, besides the diagnosis codes included in the three definitions, spindle cell sarcoma (8801/3) in some practices may potentially be UPS cases since it is a rather catch-all term in clinical practice. If it was included as UPS in the third definition, the incidence would further increase (up to 0.76 per 100,1000) and account for a higher proportion in all STS (up to 24.8%). The other limitation of this study is that SEER22 registries covered not all but about 48% of the US population. It is possible that our estimates may not be representative and underestimate the entire US population as the diagnosis and recording of UPS could vary by region and clinical practice. Based on the data we have presented, UPS appears to represent a significant number of patients in the US and warrants attention and further research of this devastating group of diseases.

## 5. Summary and Conclusions

In summary, the incidence of UPS continued to decrease in the past 2 decades in the US. However, it is challenging to estimate the real incidence of UPS due to potential confusion in its definition and classification. Guidelines in UPS diagnosis and classification need to be better implemented throughout clinical practices in the US.

## Figures and Tables

**Table 1:** Histologic types and ICD-O3 codes for soft tissue sarcomas.

Histologic type	ICD-O3 code
Fibrosarcomas	8810–8813
Infantile fibrosarcoma	8814
Fibrous histiocytoma, malignant	8830
Dermatofibrosarcomas	8832–8833
Liposarcomas	8850–8855, 8857
Leiomyosarcomas	8890–8891, 8894–8895, 8897
Rhabdomyosarcomas	8900–8902, 8912–8921
Embryonal rhabdomyosarcoma	8910
Hemangiosarcomas	9120, 9124, 9170
Hemangioendothelioma, malignant	9130
Hemangiopericytoma, malignant	9150
Kaposi sarcoma	9140
Malignant peripheral nerve sheath tumor	9540
Malignant Neurilemmoma	9560–9561
Neuroblastomas	9490, 9500–9503, 9505–9506
Malignant granular cell tumors	9580
Synovial sarcomas	9040–9043
Malignant mesotheliomas	9050–9054
Giant cell and extraskeletal bone sarcomas	9180–9181, 9220, 9231, 9240–9243, 9250–9252, 9260
Chordomas	9370–9372
Myxosarcomas	8840–8841
Sarcomas, NOS	8800–8803
Stromal cell sarcomas	8930–8931
Alveolar soft part sarcoma	9581
Epithelioid sarcoma	8804
Clear cell sarcoma, NOS	9044
Complex mixed and stromal neoplasms	8935–8936, 8964
Miscellaneous other sarcomas	8805, 8858, 8860, 8896, 8990–8991, 9133, 9161, 9182–9187, 9192–9194, 9200, 9221, 9330

*Note:* Primary cancer site restricted to connective, subcutaneous, and other soft tissues (ICD-O-3 site code: C49.0–C49.9).

**Table 2:** Incidence of UPS by 3 different definitions∗ during 2016–2020, SEER22.

Definitions	Incidence (1/100,000)		Proportion of UPS in STS (based on crude incidence) (%)
	Crude	Age-adjusted	
1. UPS (8830)	0.06	0.05	1.8
2. UPS (8830, 8810, 8811, 8813, 8840)	0.38	0.34	11.2
3. UPS (8830, 8810, 8811, 8813, 8840, 8800)	0.67	0.61	20.0

^∗^UPS patients were identified using three different definitions: (1) MFH only (ICD-O-3 histology code: 8830); (2) MFH plus fibrosarcoma NOS, fibromyxosarcoma, fascial fibrosarcoma, and myxosarcoma (8830, 8810, 8811, 8813, 8840); and (3) definition 2 plus sarcoma NOS (8830, 8810, 8811, 8813, 8840, 8800).

## Data Availability

The data used in this study are from SEER, a public available database (https://seer.cancer.gov/data/access.html).

## References

[1] Toro J. R., Travis L. B., Wu H. J., Zhu K., Fletcher C. D., Devesa S. S. (2006). Incidence Patterns of Soft Tissue Sarcomas, Regardless of Primary Site, in the Surveillance, Epidemiology and End Results Program, 1978–2001: An Analysis of 26,758 Cases. *International Journal of Cancer*.

[2] Goldblum J. R. (2014). An Approach to Pleomorphic Sarcomas: Can We Subclassify, and Does it Matter?. *Modern Pathology*.

[3] Sbaraglia M., Bellan E., Dei Tos A. P. (2020). The 2020 WHO Classification of Soft Tissue Tumours: News and Perspectives. *Pathologica*.

[4] Horner M. J. R. L., Krapcho M., Neyman N (2006). *SEER Cancer Statistics Review*.

[5] Howlader N. N. A., Krapcho M., Miller D (2018). *SEER Cancer Statistics Review*.

[6] Fletcher C. D. (2014). The Evolving Classification of Soft Tissue Tumours—An Update Based on the New 2013 WHO Classification. *Histopathology*.

[7] Gusho C. A., Lee L., Guntin J., Blank A. T. (2022). Comparison of Features and Outcomes of Undifferentiated Pleomorphic Sarcoma of Bone and Soft Tissue. *Journal of Surgical Research*.

[8] Xu F., Zhao F., Feng X (2021). Nomogram for Predicting Cancer-Specific Survival in Undifferentiated Pleomorphic Sarcoma: A Surveillance, Epidemiology, and End Results-Based Study. *Cancer Control*.

[9] Brennan M. F., Antonescu C. R., Moraco N., Singer S. (2014). Lessons Learned From the Study of 10,000 Patients With Soft Tissue Sarcoma. *Annals of Surgery*.

[10] Penel N., Coindre J. M., Giraud A (2018). Presentation and Outcome of Frequent and Rare Sarcoma Histologic Subtypes: A Study of 10,262 Patients With Localized Visceral/Soft Tissue Sarcoma Managed in Reference Centers. *Cancer*.

